# Doctors and Quacks*The spirited editorial of our *confrère* of the London Medical Times and Gazette finds a happy application in our western world, and deserves the reader’s atention.—*Ed. Virginia Med. Jour.*

**Published:** 1858-11

**Authors:** 


*Doctors and Quacks.**—A clever and pungent writer, who in the Saturday Review often hits the right nail pretty sharply on the head, has lately told us a word or two of his mind about "doctors and quacks." We call attention to what he has written on this topic, because his opinion on medicine and its practitioners seem to us to represent indifferently well the opinions of many of the best informed and most reasonable of the non-professional public on these matters. There is, it is imposible to deny the fact, some truth mixed with a large share of fallacy in these opinions; and we would gladly, therefore, endeavor, while admitting what is true, to explain to candid minds wherein lie the errors of the conclusions which are there accepted as undeniable facts.

The writer tells us that the languor exhibited by parliament on matters connected with medical reform is not attributable solely to the difficulty of reconciling conflicting interests. There is a feeling pretty widely distributed, he asserts, among the public, and extensively felt even in the house of commons, that the sick get well when not treated "according to received rule;" and there is a fear lest these reform bills should be used "to annoy every

*The spirited editorial of our *confrère* of the London Medical Times and Gazette finds a happy application in our western world, and deserves the reader's atention.—*Ed. Virginia Med. Jour.*

one who is suspected of disaffection to the creeds and symbols of the drug dispenser."

The great fault of doctors, he goes on to say, is this, that they ignore contemptuously all new plans of treatment, and all systems of medicine which do not square with their idea and standard of orthodoxy; but what is the use of this? "Almost every body in private society has of late years had friends who left them sinking under disease to make trial of some of the tabooed systems, and then returned in ruddy health; and yet so strong are known to be the prejudices of medical men that it is thought hardly civil to mention a case of the kind in their presence." And when doctors allude to these systems, it is only to ridicule them, or to vilify and vituperate their authors; they never condescend to examine and investigate the truth or fallacy of them, like honest observers. "The English medical profession includes knowledge of the highest, and talent of the most various kind;" and yet their abuse is like that of theologians in by-gone days "when theologians were not as meek and peaceable as, of course, they are at present. The medical press is the most scurrilous in England, and its personalities reduce to insignificance the gentler venom of religious journalism," writes the Saturday Review!

"It is not, however, of the physician that the public can complain; as usual, it is the democracy of the profession, the body of general practioners, which is really and sternly exclusive."

Now, when we plainly and honestly endeavor to explain the injustice and fallacy of these accusations, we are always most unfairly stopped by that unanswerable tu quoque rejoinder, "Ay! you deny all this, of course—it don't suit your book to admit it; it's contrary to your creed; we know the value of your arguings and facts before you utter them. What you have to say is the old tale all over again." And the actual truth is, that we are compelled to repeat the old tale over and over again, and simply for this reason, that the old tale is a true, honest, and scientific representation of the facts it has to deal with. Time, which squares all anomalies, will one day do us justice; and, in the mean time, all that remains for us is to lay the honest bare truth of this matter, as we know it, before the world, trusting and believing that there are some candid and discriminating minds, who are able to appreciate the difficulties and yet recognize the solidity of our position, when it is pointed out to them.

Let us then argue these points calmly, and as rational beings should address each other. Don't let us commence the argument by accusing each other of scurrility, for example; for by so doing we only convict ourselves of being in possession of the beam, which we think we see in our neighbor's eye. Let us honestly endeavor to find out the causes which provoke so many of the world to express their dissatisfaction with medicine as it is, and to fly into the open arms of quackery. By so doing we shall be enabled to answer the question you ask; What is the definition of a quack? And we shall also, perchance, ascertain at whose account lies the blame of all these evils.

Now there are, as we see the matter, two distinct provoking causes, acting in different directions, which have brought so many of the world to this dislike and positive distrust of the medical profession, and have induced so many to carry on a war of proselytism against it.

One cause is to be found in the conduct of the medical profession itself; with the other, the public is distinctly chargeable. Let us now, first of all, contemplate the conduct of those who cast these stones at our profession: let us see if it is consistent with common sense and common wisdom. You admit, "that a man must be a fool who does not wish that his medical attendant should have as much as possible of the knowledge, skill and powers of observation which constitute the superiority of a Brodie, a Holland, a Clark, or a Watson. But, you add, "when we come to strictly curative methods and appliances, the feeling is visibly different." Now, what does this mean, if it have any sense but this: that the deeper a man is grounded in a scientific knowledge of his profession, the less you, the public, judge him to be capable of practicing it. Yes! you, the voice of the public wisdom, accept this manifest absurdity. And when you speak of the thoughts and sentiments of the public and of the house of commons, did it never occur to you to ask yourself the worth and value of the thoughts and sentiments of those bodies about the question in hand? Did it never strike you that the public may be possibly a very ill judge of the matters it has taken upon itself to decide? Have you forgotten that you are dealing with a subject on which, as far as the public is concerned, men's faith, rather than their reason, is brought into exercise; and have you not the proofs lying all broadcast about you, how grossly superstitious this said public never misses an opportunity of proving itself to be?—ay! and the learned, the educated, the high-placed public too? Witness its million antics in every age; and in our own, think of metallic tractors, St. John Long, mesmerism, clairvoyance, spirit-rapping, homœopathy, table-turning, and the other thousand and one mad follies of the day. What made Michael Farraday boldly tell you, the educated public of the land, that poor education was defective, but this very fact, that he found, when the hour of trial came, you succumbed; that your minds were not strengthened by that knowledge of physical science which would enable you to avoid the degradation of being drawn into the vortex of every passing folly of the day.

And if you want an unanswerable proof of the ignorance and superstition of that public which pretends to be our judges, of its amazing credulity, look at the broadsides of seven-eighths of the newspapers of this country! You, the journalists of the country, accuse us of scurrility! You are angry with us for telling the truth, because we denounce, in no courtly terms, the bestialities which, as advertisements, disgrace and disfigure your journals. You know that the shameful trade of torture and robbery carried on by quackery, through the aid of the press, is the main support of half the journals of this country. Advertisements figure on those journals which you would not permit your wife or child to read; and yet, ponder it well, those filthy things keep your journals alive. One advertising firm alone, we have heard it credibly asserted—one firm alone of that class in this metropolis spends from £12,000 to £15,000 a year upon your journals!* You ask for a definition of quackery, and we tell you to read the advertisements on the back of your journals, and you will see one and the worst part of it

*Of course we gladly acknowledge that there are journals who do not thus prostitute their pages, but these are only the "rari nantes." The truth remains unanswerable, that the large body of the press of this country is perverted to this most unholy business.

depicted there. In the face of such abominations as these, can you expect a man who knows their meaning, and their purport, and their effects, to be nice and genteel and mealy-mouthed in his language? And can you be surprised if we wish to dissociate ourselves as utterly as possible from all connection with the authors of those advertisements? Why should not our colleges and universities have the power, equally as the bar, and as the army, and as the church, of ridding the profession of (or persecuting, as you call it) a member who had disgraced it. Let him carry on his trade if he choose, but not under the sanction of an honorable title.

Thus it is, then: you, the public, who spend millions a year in the encouragement of this foul and manifest deceit and quackery; you, the governing body of the country, who give the stamp of your approbation to every nostrum that desires it—you pretend to be the judges between the medical profession and quackery! Are you just, reasonable, or even honest.

When you accuse the profession of not explaining or admitting the wonderful cures and recoveries which are continually taking place outside the profession, you are accusing them of intangible crimes. An explanation of such cases, whenever their whole history can be got at, which would satisfy a scientific physician, would be incomprehensible to the public mind, especially when that mind is already made up—when the faith has preceded the reason of the thing. You know well enough, that in all these cases, you ask us for explanations, not for the sake of getting at the truth, but simply to glorify your disease-curer; you know well enough, that before we open our mouths your mind is irrevocably made up on the subject. However, we would like under this head, to ask one question of those who throw these wonderful cures in our teeth—is it, or is it not true, that ninety-nine hundredths of the real diseases of the people of this country are treated by the medical profession, and that such diseases, so treated, if not cured by their medical skill, remain, and are incapable of cure? Why should these miraculous cures you speak of, if really true ones, be instanced only in some few cases striking to you and yet intangible to us? Why not prove the truth of your conjurer's position, by getting him to cure, not one, but a whole class of diseases, which we confess and admit to be beyond the reach of our skill?

The real fact is that you—the public—are not only, as we have proved by your doings, incapable of judging in this matter, but you are, moreover, unable and unwilling to hear and admit the honest truth, when it is honestly told you. Let a physician tell his patient the clear truth, the truth such as a long and arduous study of scientific medicine justifies him in telling—as, for example, that his patient's disease is of a nature which is little amenable to any mere ordinary medical agencies, or that it is incurable; let him do so, and he will, in all probability, never see the patient again. Let the patient be told the same thing by half a dozen equally skilled observers, and yet he will remain unsatisfied, and is almost certain to find his way into the hands of the most ignorant advertising empiric, who will promise him all he desires, and of course falsely promise him. The profession meet with these cases every day of their lives; but you—the public—hear nothing of the failures. One happy hit will make the fortune of a quack—a thousand failures do him no harm with the public; the one is widely bruited to the world, the others are buried with the bones of the patients. The public are most unreasonable; they expect, on all occasions, from the medical art and from medical skill what neither the one nor the other can on all occasions accomplish for them; they are impatient if told that they cannot be so readily rid of their ailments; and then they rush to where they can be gratified by the sounds of delusive promises. They love this deception, and herein lies the exact difference between medicine and quackery—the one promises what it can perform, the other what it cannot. It is an unholy impatience of bearing the necessary ills which "flesh is heir to," that makes so many deride true medicine, and fly to the delusions of knavery.

We have spoken now but one phasis of quackery—of that disreputable advertising division of it of which you yourself, I will suppose, do not undertake the patronage; which you as reputable journalists do not even permit to gain reputation through your columns, and which, therefore, as the higher portion of the press, you stamp as disgraceful. And now we ask you to explain this glaring anomaly; that you pass over in silence this monster evil, which you know and admit is degrading the minds and injuring the bodies of thousands of your countrymen, which is yearly fed by millions of the money of the ignorant—that you have no word of reprobation to say to those of your fellow journalists who live by and promote this evil, but save all your anger, and pour out your ill-will, upon a body of men who have received a deeply-scientific education, and who, in honor, indignantly exclaim against the abominations which their knowledge forces them to loathe. You act thus, and then call us bigoted, and proclaim yourselves the fit and proper judges of the value of the scientific knowledge of a medical man!

